# Is There an Association between the Gingival Phenotype and the Width of Keratinized Gingiva? A Systematic Review

**DOI:** 10.3390/dj9030034

**Published:** 2021-03-23

**Authors:** Elpiniki Vlachodimou, Ioannis Fragkioudakis, Ioannis Vouros

**Affiliations:** Department of Preventive Dentistry Periodontology and Implant Biology, Dental Faculty, School of Health Different Sciences, Aristotle University of Thessaloniki, 54624 Thessaloniki, Greece; elpiniki.vlachodimou@gmail.com (E.V.); ifragkio@gmail.com (I.F.)

**Keywords:** gingival biotype, gingival phenotype, gingival thickness, width of keratinized gingiva, periodontal biotype, gingival periodontal biotype/phenotype, periodontal biotype and width of gingival, periodontal disease and biotype, thin and thick biotype

## Abstract

The concept of gingival phenotype and width of keratinized gingiva influencing the diagnosis and treatment in the periodontal scenario is relatively new. Soft and hard tissue dimensions of oral tissues are considered essential parameters in daily clinical practice. Factors such as the biotype category and the width of the keratinized gingiva help dentists seek the perfect therapy plan for each patient to achieve long-term stability of periodontal health. Several methods have been proposed to categorize phenotypes and each phenotype is characterized by various clinical characteristics. This review aims to discuss the possible association between the gingival phenotype and the width of keratinized gingiva along with the results appeared. After a rigorous search in major electronic databases, the results of the included studies indicated that the width of keratinized gingiva seems to be associated with the periodontal phenotype, with thick biotypes being characterized by a more pronounced keratinized gingival width. However, the heterogeneity of the included studies did not allow to make a conclusion about a direct relationship.

## 1. Introduction

Over the last years, it has been widely accepted that gingival width and thickness vary not only among different individuals but also in various positions in the oral cavity [1,2]. The shape, consistency, and position of the gingiva and the alveolar process define each individual’s specific “periodontal biotype”, affecting the periodontal response to inflammation and its treatment [3]. The genetically determined type of gingiva is defined as a “periodontal biotype” and characterized by the gingival thickness (GT) and a specific morphology, as well as the underlying alveolar bone [4]. Many authors have labelled the “biotype” with various names such as, “gingival” or “periodontal”, “biotype”, “phenotype”, or “morphotype”. Nevertheless, according to the 2017 Classification of periodontal diseases and conditions in the World Workshop of Periodontology, the term “biotype” has been changed to “phenotype” and using the new terminology, in this review, it will be referred to as “periodontal phenotype”. Moreover, determining the periodontal phenotype is often challenging since the typical forms of biotypes are found in a small percentage of individuals. At the same time, the majority of people have an intermediate clinical appearance [5].

Nowadays, we can distinguish between three types of gingival phenotype: thin scalloped, thick flat, and moderate [2]. The latter is a recent addition to the categorization of periodontal phenotype, as it presents a clinical manifestation of an intermediate form with some characteristics of the other two types. The most popular biotype categorization methods used in contemporary clinical practice are the transparency (TRANSP) of the periodontal probe through the gingiva sulcus [6] and the transgingival probing method (TRANS) [7]. Both of these techniques are based on gingiva thickness to determine the phenotype and do not take into account the underlying bone thickness or other characteristics. In other words, modern phenotype classification methods use a variety of criteria, and an accurate determination cannot always be achieved.

The keratinized gingiva width (WKG) is defined as the distance between the mucogingival junction (MGJ) and the gingival margin [1]. It has been suggested in the past, that to ensure periodontal health, the width of the keratinized gingiva should be at least 2 mm while the attached gingiva should measure 1 mm [8]. Besides, a minimum of 5 mm of keratinized gingiva has been proposed to ensure proper subgingival restoration margins [9]. Nowadays, it is well-documented that, despite the lack of keratinized tissue, periodontal health can be maintained when adequate oral hygiene measures are applied by the patient [10].

It is essential to mention that these two factors, periodontal phenotype and width of keratinized gingiva, constitute essential tools to plan a successful periodontal, prosthetic, and orthodontic treatment since the different reactions of each phenotype to inflammation and surgical trauma can modify the treatment decisions. In case a correlation between these two factors would be established, they may be considered as significant tools with a view to organizing the treatment plan. However, there is a lack of information regarding the association among these two parameters, as only a few clinical studies have investigated their relation. Taking the above into account, a search for evidence supporting the possible association between these two parameters would enable the selection of a predictable treatment plan (Cortellini and Bissada, 2018a).

The present systematic review aims to investigate the correlation between gingival phenotype (GP) and the width of keratinized gingiva (WKG) based on the systematic appraisal of data provided by primary clinical studies.

## 2. Materials and Methods

This systematic review was performed according to the recommendations and principles of the PRISMA statement, an evidence-based minimum set of items for reporting in systematic reviews and meta-analyses. Prior to its conduction, a comprehensive protocol was developed and accordingly approved by all authors. This detailed protocol incorporated several sections and research techniques, such as the search strategy, definition of eligibility, inclusion/exclusion criteria, data extraction, quality assessment, and data synthesis/analysis.

### 2.1. Focused Question

To satisfy the primary aim of the study, the following focused questions were created: What are the characteristics of various forms of periodontal phenotypes, what are the critical anatomic dimensions of the involved soft tissues, and what is the relation between the periodontal phenotype and the width of keratinized gingiva?

### 2.2. Search Strategy and Study Selection

The search strategy incorporated the searching of electronic databases, supplemented by cross-checking bibliographies of relevant review articles. A search in The National Library of Medicine, Washington, DC (PubMed–MEDLINE) was conducted up to and including the 30 May 2020. The search was designed to include any published paper to identify any appropriate study relevant to the main aim of the review. The following keywords were used in the search: the population, intervention, comparison, and outcome (PICO) framework was used to guide the inclusion or exclusion of studies for the question using the following criteria.

The following keywords were used to define our population selection:

i. Population: “healthy adults” OR “periodontal health” OR “general population” OR “epidemiological” OR “maintenance” OR “clinical study” OR “human study” OR “recall”;
ii. Intervention: types of studies, e.g., “randomized controlled trials (RCTs)” OR “cohort studies” OR “case-control clinical studies” OR “cross-sectional studies” OR “qualitative studies”;
iii. Outcome: “gingival/periodontal biotype” OR “gingival/periodontal phenotype” OR “width of keratinized gingiva” OR “gingival width” OR “gingival thickness” OR “gingiva’s dimensions” OR “thick/thin biotype”.

These terms were then combined as follows: population/exposure AND outcomes, association/correlation AND intervention.

In addition, the bibliographies of relevant review articles were screened for inclusions and references of all included publications were screened for further relevant studies. Moreover, a hand search was conducted. The following journals were considered potentially significant and were hand-searched: Journal of Clinical Periodontology, Journal of Dental Research, Journal of Periodontology, Journal of Prosthetic Dentistry, British Dental Journal, International Journal of Periodontics and Restorative Dentistry, International Journal of Prosthodontics, Quintessence International, Periodontology 2000, Acta Odontologica Scandinavica, Journal of Periodontal Research.

### 2.3. Inclusion/Exclusion Criteria

According to the above observations, the inclusion criteria are summarized in the following:

i. randomized clinical trials (RCTs), controlled clinical trials (CCTs), cohort studies, prospective or retrospective clinical studies, and original cross-sectional studies reporting on individuals with >20 teeth;
ii. included studies should present a minimum number of 20 patients;
iii. participants with periodontal health; absence of systemic diseases associated with gingiva’s manifestations;
iv. intervention: in order to be included, studies had to report on biotype/phenotype assessment with classical methods (De Rouck, Kan) as well as WKG evaluation and a possible correlation among the two parameters (GP and WKG);
v. the included clinical studies should report on data related to the width of keratinized gingiva and gingival thickness by utilizing quantitative methodologies.

Exclusion criteria included (1) patients presented with periodontal disease; (2) case reports case series; (3) clinical studies with <20 participants; (4) ex vivo and animal studies.

### 2.4. Screening Process and Data Extraction

A screening process was performed independently and in duplicate by two of the authors (E.V. and I.F.) to increase the relevance of the extracted data. All titles resulting from the initial search were screened to exclude irrelevant publications, such as review articles, and/or animal studies (E.V. and I.F.). Afterwards, an abstract screening of the remaining articles took place (E.V. and I.F.). The final stage of screening involved full-text reading and was performed by the same two reviewers (E.V. and I.F.) using a predetermined data extraction form to confirm the eligibility of each study based on inclusion and exclusion criteria. The level of agreement between the reviewers for potentially relevant articles was assessed by kappa statistics for all steps of the screening process (K = (0.960.4 − 0.9322)/(1 − 0.9322) = 0.4159 or 41.59%). During each stage, any disagreement was resolved by discussion, and in case of conflicts a third reviewer was consulted (I.V.). If a consensus regarding the inclusion of an article was not achieved, the article was included in the next stage of screening (Table 1).

**Figure 1:**
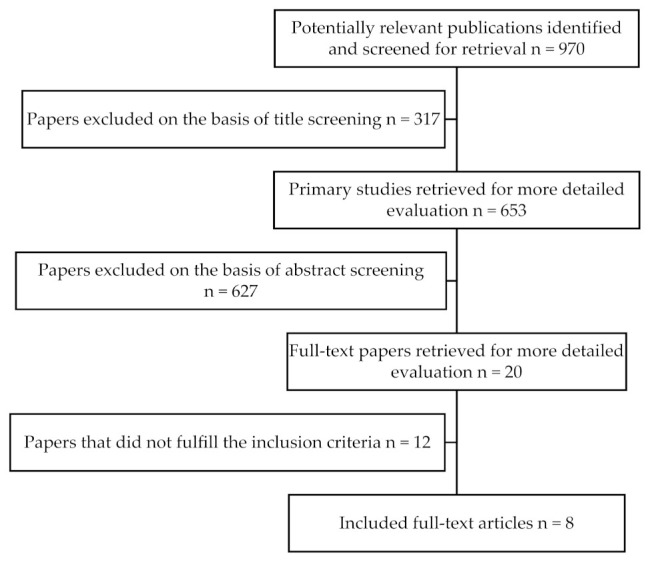
Flowchart diagram of the search strategy.

The flow chart describing the screening process as well as the selection of the appropriate articles is depicted in Figure 1.

### 2.5. Quality Assessment

A quality assessment of all included studies was performed independently and in duplicate by the two reviewers (E.V. and I.F.) and during the data extraction process. The Newcastle–Ottawa Scale (NOS) was utilized for the methodological quality assessment of the included studies. The NOS system contains eight items, categorized into three dimensions including selection, comparability, and depending on the study type outcome (cohort studies) or exposure (case-control studies). For each item, a series of response options are provided. A star system is used to allow a semi-quantitative assessment of the study quality such that the highest quality studies are awarded a maximum of one star for each item with the exception of the item relating to comparability that allows the assignment of two stars. A maximum of nine stars can be allotted if all the above items are satisfied (Table 2).

## 3. Results

The purpose of this study was to investigate the association between the gingival phenotype and the width of keratinized gingiva following the steps for a systematic review. Eight articles satisfied the inclusion criteria. To be more specific, these eight studies associating periodontal phenotype and width of keratinized gingiva frequently involved healthy patients with a clear medical history and periodontally healthy conditions, while factors, such as age, bone quality, systematic diseases (e.g., diabetes mellitus, osteoporosis), medications (e.g., anticoagulant medications, long-standing steroid medication), radiotherapy, and chemotherapy were acting as confounding factors (Table 1). Most studies used the transparency through the sulcus method (TRANSP) or the transgingival probing method (TRANSG) to categorize the phenotype, while the WKG was measured mostly as the distance between the gingival margin and the MGJ. In some studies, three or four different phenotype categories where used, while in others a direct association among phenotype and WKG was not recorded; instead an association among the GT and WKG was examined. In addition, there was high heterogeneity among the studies regarding the parameters examined, since in most of them, the WKG was studied as a secondary variable (Table 3). All studies concluded to specific outcomes (Table 4) and based on the above articles, there is evidence on establishing a distinct association between the gingival phenotype and the width of the keratinized gingiva.

More specifically, recent trials found a moderately positive association among the examined factors using the TRANSP technique [1,2,3]. However, though in the study by Di-Jing the phenotype was categorized as thick or thin, De Rouck and colleagues distinguished three distinct patient clusters regarding characteristics, such as GT, crown width to crown length (CW/CL) ratio, and WKG [1,3]. In addition, Fisher et al. used a prototype double-ended probe leading to three distinct phenotype groups (thin, thick, and moderate) [2]. Studies employing the TRANSG method confirmed the previous results. Two more studies, using the transgingival probing method categorized the gingival phenotype as thick and thin or as thick-flat, average-scalloped, average and thin-scalloped [4,5]. In the latter study, a CBCT was also used in order to evaluate the reliability of the results. The statistical analysis revealed a statistically significant correlation among the periodontal phenotype and the width of keratinized gingiva, concluding that patients with thinner gingiva frequently presented with a limited amount of attached gingiva. Furthermore, a recent systematic review reported in 2020 studying the effect of periodontal phenotype on periodontal health, showed that there was a positive correlation between the gingival thickness and WKG in maxillary anterior teeth, while the gingival phenotype did not appear to be influenced by other parameters such as age or sex [6]. Moreover, the authors concluded that the identification of the gingival phenotype is essential so as to ensure periodontal health and for planning a restorative or orthodontic treatment, especially in areas where there is thin and narrow gingiva tending to have more recession compared with those with thick and wide gingiva [6].

In contrast, a few studies did not succeed in establishing an association between WKG and gingival phenotype as no statistically significant correlation was found [7,8]. In both studies, the phenotype at the maxillary teeth was recorded by using the transparency of the periodontal probe through the sulcus (TRANSP). In the latter, the labial bone thickness was also evaluated radiographically. The findings were not in line with the previous studies and did not seem to support a clear association between WKG and phenotype. Furthermore, the presence of a moderately low association between gingival phenotype and WKG has been found in a recent study [9]. In this trial, several widespread methods were used to assess the gingival phenotype, such as direct measurement, ultrasonic device, cone beam computed tomography [10], and probe transparency through the free gingiva. This is in agreement with Zweers, indicating a positive association between keratinized tissues and gingival phenotype [21]. Nevertheless, according to the results, a relatively low to medium association among WKG and GT was found [21].

## 4. Discussion

As already indicated, the periodontal phenotype is characterized by specific anatomic characteristics, such as the gingival thickness (GT), the gingiva width (WKG), and the bone morphotype (BM) [22]. Considering the width of the keratinized gingiva, there is evidence that a thick phenotype is combined with a large amount of keratinized gingiva. To be more specific, according to Cortellini (2014), the calculated mean value, which is related to the thick biotype was 5.72 (0.95) mm (95% CI 5.20; 6.24) and regarding the thin biotype, it was 4.15 (0.74) mm (95% CI 3.75; 4.55) [22]. Nevertheless, a direct association has yet to be proven.

More specifically, in the majority of the studies, a thick gingival phenotype is associated with a wider band of keratinized gingiva. The present systematic review tried to provide more solid evidence on that hypothesis. It is worthy to mention that there are factors related to this association, which have not been clarified yet. For example, the existence of three or more different phenotype categories, rather than the traditional two types as proposed by Olsson and Lindhe. The term “periodontal biotype” was introduced by Seibert and Lindhe in 1989, who described two different categories, the thin-scalloped and the thick [23]. In 1993, Olsson and colleagues introduced the term “periodontal morphotype” after observing that teeth with long clinical crowns are more prone to develop gingival recession [24]. Since then, many scientists have studied periodontal biotypes and proposed various categorization methods. A few years later, Aimetti and co-workers defined the periodontal morphotype as thin (<1 mm) or thick (>1 mm) [25]. Additionally, Kan described thick gums as dense and fibrous in appearance while the thin ones should be more brittle and almost transparent [26]. However, we should not fail to mention that this strict classification of biotypes into two groups does not include extreme cases presenting characteristics quite different compared to those of the groups originally classified [7].

According to the classification of periodontal biotype/phenotype, as defined in 2017 in the World Workshop of Periodontology, two factors were proposed for the assessment of biotype categorization:

A. the gingival phenotype which describes the morphology of the periodontal tissues and includes the thickness of the gingival tissues and the width of the keratinized gingiva

B. the bone morphotype, concerning the thickness of the buccal bone plate [27].

In addition to these two factors, the size and shape of teeth are also evaluated to distinguish the phenotype. The new classification constitutes three categories of periodontal phenotypes:

A. thin-scalloped: associated with a slender and triangular tooth crown, small dental cervical curvature, points of contact to the incisal surfaces, the narrow width of keratinized gingiva, thin gingiva at the CEJ, and relatively thin labial bone plate at both distances apical to the CEJ;

B. thick and flat: more square teeth with marked cervical curvature, large contact areas between the teeth located more apically, a wide range of keratinized gingiva, thick fibrous gingiva, and relatively thick alveolar bone sheath;

C. thick-scalloped: thick fibrous gingiva at CEJ, thin teeth, quadratic tooth form, narrow width of keratinized gingiva, highlighted wavy contour of the gingiva, thick labial bone plate [27].

Recent studies, in which cone beam computed tomography (CBCT) was used, proposed two more types of periodontal phenotype. As a result, an average form and a mixed type were identified and their characteristics were described.

Since each study used different criteria to categorize the gingival phenotype, the frequency distribution between biotypes varied in our population. In general, a thick phenotype (51.9%) is more frequently observed than a thin phenotype (42.3%) when assessed based on gingival thickness [22]. According to a systematic review, the majority of studies up to 2013 adopted the classification of gingival phenotype based on the classic study of Olsson and Lindhe [28], which categorized gingival biotypes into thin or thick, avoiding the intermediate category of medium thickness [11,26,29].

Estimating the dimensions of soft tissues is pivotal in determining the periodontal phenotype category [21]. There are several methods to discriminate biotype categories including direct techniques or even ultrasound [26].

A clinical method, which Kan and colleagues suggested in 2003 [30], is based on the periodontal probe’s transparency through the mucogingival junction (probe transparency method, TRANSP). The accuracy of this technique was confirmed by De Rouck in his study in 2009. If the probe’s contour is visible through the gingival margin, the gingival phenotype is characterized as thin. Otherwise, the phenotype is considered thick [3]. This is a qualitative distinction of gingiva as it is not based on an assessment of the soft tissue’s thickness. Kan and colleagues in 2010 used a transgingival probing method (TRANSG) and defined the limit of 1 mm to measure the thickness of gingiva [26]. If the thickness is measured below 1 mm, the gingival phenotype is considered thin. More specifically, they compared the probe transparency method with the method which measures the gingival thickness from the edge of the free gingiva. In this method, the probing limit to measure the gingival thickness was defined at 2 mm and it was implemented in the middle of the buccal surface of the upper anterior teeth. As a result, they concluded that, when the thickness of the gingiva was less than 0.6 mm, the gingival phenotype was suggested in all cases thin and, when the thickness of the gums exceeded 1.2 mm, then the phenotype was always considered thick. The researchers arbitrarily proposed the limit of 1 mm for the gingival thickness as a criterion for distinguishing between the two biotypes based on the above data [26].

In 2015, Frost and his partners tried to determine the exact limit of gingival thickness to classify the biotype as thin or thick, by using the probe transparency method [31]. For this purpose, they measured the thickness of gingiva in 306 anterior teeth of the upper jaw in 56 dental students, using an endodontic file with a diameter of # 30 according to ISO, which was placed perpendicular to the attached gingiva at a distance of 2 mm from the edge of free gingiva. At the point where the endodontic file met the surface of the gingiva, liquid resin was applied, followed by polymerization. The file’s length was measured with a digital caliper, and the participants’ biotype was classified as thick or thin. The results showed that it is impossible to determine a specific value of gingival thickness to help us classify the biotype. The authors concluded that measuring gingival thickness is not recommended for biotype determination.

The probe transparency method (TRANSP) is closely related to high reproducibility (85%) by various operators [3]. This is why this technique is considered the gold standard in clinical practice [31] as scientists, for the most part, apply it in their clinical studies. According to other researchers, this method cannot distinguish the periodontal biotype if the gingival thickness is not greater than 1.2 mm or less than 0.6 mm [26,32].

The lack of homogeneity among the studies may have yielded contradictory results, as all the available studies are quite heterogeneous in terms of the study population, design, or biotype discrimination methods. Nevertheless, even if a direct correlation between the phenotype and the width of the keratinized gingiva may not be supported by the existing data every time, the possibility of an existing positive association would enhance clinical procedures.

The identification of the periodontal phenotype may be necessary for clinical practice since differences in gingival and osseous architecture are related to the outcome of different therapeutical procedures, including periodontal treatment. In the early 1990s, Olsson and Lindhe demonstrated that periodontal inflammation would affect different phenotypes inconsistently, resulting in deep periodontal pockets in a thick and a gingival recession in a thin scalloped biotype [28]. Therefore, non-surgical periodontitis treatment would lead to a significant gingival recession in patients with a thin phenotype compared to patients with a thick periodontal phenotype [33]. Accordingly, the outcome of a surgical approach in the periodontium could also be influenced by the periodontal phenotype. Pontoriero and Carnevale (2001) showed, in a study investigating the treatment outcome after crown lengthening procedures, a more notable soft tissue regaining in patients with thick periodontal phenotypes compared to thin periodontal phenotypes. Hence, if we discuss mucogingival surgery and root coverage techniques, the initial thickness of keratinized soft tissues was found to be associated with complete root coverage when using a coronally advanced flap [34]. A critical threshold of gingival thickness >1.1 mm has been reported when complete root coverage is acquired in root coverage procedures [35].

Another critical issue related to the above association is orthodontic therapy planning. In cases with a thin phenotype, dehiscence and/or fenestration, leading to gingival recession may occur if the tooth movement is directed outside the osseous housing [36]. A careful radiographic examination by means of computer tomograms and even a pre-orthodontic soft tissue augmentation may be needed in cases with a thin phenotype. In addition, in implant therapy, the periodontal phenotype has been described as a decisive factor for the success of treatment outcomes [12,30,37]. A tendency for gingival recession was found after immediate single implant placement in patients with a thin scalloped phenotype [38], while a reduced risk of soft peri-implant tissue recession was observed in patients with a thick phenotype [39]. In other words, a clinician must acknowledge the biotype before any treatment planning in the field of restorative dentistry.

## 5. Conclusions

- According to the findings of the present systematic review a positive correlation between the width of keratinized gingiva and the periodontal phenotype seems to be established.
- Gingival thickness constitutes the key parameter linked to the two parameters (WKG and periodontal phenotype), with thick biotypes being characterized by a more pronounced WKG.
- The width of keratinized gingiva and periodontal phenotype are considered as valuable parameters that can influence the outcome of various treatment modalities in periodontal and restorative dentistry.
- The thin phenotype constitutes a risk factor when a treatment plan is established in the fields of periodontology, restorative dentistry, and orthodontics and, as a result, patients showing such characteristics should be very carefully approached in this respect.
- However, it should be noted that the existing data in this respect are scarce, which leads to the fact that more clinical studies with a homogenous design are needed to provide substantial evidence for the association between gingival biotype and the width of keratinized gingiva.

## Figures and Tables

**Table 1:** Excluded studies and reasons for exclusion.

Excluded Studies	Reasons for Exclusion
Xu et al., 2015 [1]	This article is written in Chinese
Cook et al., 2011 [11]	No association among biotype and WKG assessed
Lee et al., 2013 [12]	No association among biotype and WKG assessed
Alkan et al., 2018 [13]	No association among biotype and WKG assessed
Fogorvosi et al., 2016 [14]	This article is written in Hungarian
Motta et al., 2017 [15]	Did not assesses WKG
Muller et al., 1997 [16]	No association biotype and WKG assessed
Park et al., 2017 [17]	No association among biotype and WKG assessed
Rasperini et al., 2015 [18]	association among biotype and WKG
Singh J et al., 2016 [19]	No association among biotype and WKG assessed
Stellini et al., 2013 [20]	Did not assess the biotype

**Table 2:** Quality assessment.

Included Studies	Selection	Comparability	Outcome/Exposure
De Rouck, 2009	***	**	****
Stein, 2013	**	**	***
Fischer, 2014	**	**	**
Shah, 2015	**	**	***
Fischer, 2017	**	**	**
Joshi, 2017	***	**	***
Shao, 2018	**	**	***
Di Jing, 2019	***	**	***

Note: score from 7–9 stars “*” has high quality, score from 4–6 stars “*” has high risk quality, score from 0–3 stars “*” has very high risk of bias.

**Table 3:** Studies examining the association among phenotype and width of keratinized gingiva.

Study	Population	Source of Study	Teeth Examined	Study Design	Assessment of Biotype (Method)—Parameters Examined	WKG Measurement Method
De Rouck, 2009	100 ♀ 50/♂ 50 (19–56 years old)	Free University in Brussels (VUB)	13–23	Cross-sectional	TRANSP—Thick (Not visible probe), Thin biotype (Visible probe)Cluster analysis for gingival morphotype based on CW/CL ratio, GT, and WKG leading	
Stein, 2013	60 ♀ 36/♂ 24 (18–61 years old)	Department of Operative Dentistry, Periodontology and Preventive Dentistry, University Hospital Aachen		Cross-sectional	TRANSP, SC, WKG CW/CL, ratio GT and labial bone thickness measured in radiographs in 9 spotsapico-coronally	Free gingival margin to MGJ
Fisher, 2014	36 ♀ 19/♂ 17 (18–35 years old)	Julius Maximilians-University Wuerzburg	13–23	Cross-sectional	TRANSP GT, PD, PH, WKG	Free gingival margin to MGJ
Shah, 2015	400 ♀ 200/♂ 200 (20–35 years old)	Bapuji Dental College and Hospital,	13–23 mid-buccal area	Cross-sectional	TRANSG/Thick (>1 mm), Thin (≤1 mm) WKG	Free gingival margin to MGJ
Fisher, 2017	60 ♀ 39/♂ 21 (19–37 yearsold)	Witten/Herdecke University	11–21 mid-buccal aspect of an upper central incisor	Cross-sectional	Transparency of a double-ended prototype probe at the left upper central incisor/Thin (Thick ending of probe not visible), Moderate (Thick ending visible, thin not visible). Thin (thin ending visible) GT, WKG	Free gingival margin to MGJ
Joshi, 2017	800 ♀ 400/♂ 400 (18–25 years old)	School of Dental Sciences, Krishna Institute of Medical Sciences Deemed University (KIMSDU), Karad, Maharashtra, India	13–23 all the maxillay teeth in the anterior sextant	Cross-sectional	TRANSP GT and labial bone thickness measured in radiographs in 6 spots apico-coronally (GT1, GT2, GT3, AT1, AT2, AT3), WKG	Free gingival margin to MGJ
Shao, 2018	31 ♀ 16/♂ 15 (18–27 years old)	College of Stomatology, Nanjing Medical University	13–23/33–43372 teeth	Cross-sectional	GT measured through TRANSG and CBCT, TRANSP also used 4 biotypes examined, Thick-flap biotype, average-scalloped biotype, average-flap biotype, and thin-scalloped biotypeCW/CL, PH, WKG, BT in CBCT	WKG—PD
Di Jing, 2019	26 ♀ 17/♂ 9 (18–34 years old)	Peking University Health Science Center	13–23 Buccal-middle side of anterior teeth	Cross-sectional	TRANSP	Free gingival margin to MGJ

**Table 4:** Included studies and outcomes.

Study	Periodontal Phenotype (P Value)	WKG (P Value)	Main Outcomes
De Rouck, 2009	<0.001	<0.001	Relative association with Biotype and GT and WKG
Stein, 2013	<0.001	<0.001	Relatively low to medium association among WKG and GT (Pearson correlation coefficients: 0.018–0.276)
Fisher, 2014	<0.0001	0.0834	Statistical comparison showed no significant association between biotype and WKG among all subjects (P = 0.0834)
Shah, 2015	**Central incisor—lateral incisor** <0.05 **Lateral incisor—canine** <0.001 **Canine—central incisor** <0.001 **Males—females** >0.05	**Central incisor—lateral incisor** <0.001 **Lateral incisor—canine** <0.001 **Canine—central incisor** 0.05 **Males—females** >0.05	A significant positive correlation observed between WKG and GT for maxillary central incisor, lateral incisor, and canine, i.e., the patients with thinner gingiva frequently present with a limited amount of attached gingiva.
Fisher, 2017	**Thin** P_25%_ 0.32 P_0_._75%_ 0.59 **Moderate** P_0.25%_ 0.58 P_0.75%_ 0.81 **Thick** P_0.25%_ 0.74 P_0.75%_ 0.94	**Thin** P_25%_ 3 P_0.75%_ 4.1 **Moderate** P_0.25%_ 4 P_0.75%_ 6 **Thick** P_0.25%_ 4 P_0.75%_ 7	WKG appeared to be directly correlated with biotype (Spearman correlation: p < 0.001; R2 0.308).
Joshi, 2017	<0.01	>0.01	No correlation found between WKG and GT2, AT1, AT2, AT3 in males and females (p ≤ 0.01) except at GT1 and GT3 (r = 0.17, 0.14) in females (p ≥ 0.01)
Shao, 2018	<0.05	<0.05	Moderate correlation among WKG and GT (p < 0.01, Spearman’s correlation, 0.3 < r ≤ 0.5)
Di Jing, 2019	<0.001	<0.001	Moderate association among biotype and WKG (Spearman’s Correlation, r = 0.544)

## Data Availability

Data available in a publicly accessible repository. The data presented in this study are openly available in PUBMED MDPI.

## Abbreviations

TRANSP	probe transparency through the sulcus
TRANSG	transgingival probing
GT	gingival thickness
WKG	width of keratinized gingiva
CW/CL	crown width/crown length
SC	gingiva scalloping
PH	papillary height
BT	bone thickness
CBCT	cone-beam computed tomography

## References

[1] Jing W.D., Xu L., Xu X., Hou J.X., Li X.T. (2019). Association between Periodontal Biotype and Clinical Parameters: A Cross-sectional Study in Patients with Skeletal Class III Malocclusion. Chin. J. Dent. Res..

[2] Fischer K.R., Künzlberger A., Donos N., Fickl S., Friedmann A. (2018). Gingival biotype revisited—Novel classification and assessment tool. Clin. Oral Investig..

[3] De Rouck T., Eghbali R., Collys K., De Bruyn H., Cosyn J. (2009). The gingival biotype revisited: Transparency of the periodontal probe through the gingival margin as a method to discriminate thin from thick gingiva. J. Clin. Periodontol..

[4] Shah R., Sowmya N.K., Mehta D.S. (2015). Prevalence of gingival biotype and its relationship to clinical parameters. Contemp. Clin. Dent..

[5] Shao Y., Yin L., Gu J., Wang D., Lu W., Sun Y. (2018). Assessment of Periodontal Biotype in a Young Chinese Population using Different Measurement Methods. Sci. Rep..

[6] Kim D.M., Bassir S.H., Nguyen T.T. (2020). Effect of gingival phenotype on the maintenance of periodontal health: An American Academy of Periodontology best evidence review. J. Periodontol..

[7] Fischer K.R., Richter T., Kebschull M., Petersen N., Fickl S. (2015). On the relationship between gingival biotypes and gingival thickness in young Caucasians. Clin. Oral Implants Res..

[8] Joshi A., Suragimath G., Zope S.A., Ashwinirani S.R., Varma S.A. (2017). Comparison of gingival biotype between different genders based on measurement of dentopapillary complex. J. Clin. Diagn. Res..

[9] Stein J.M., Lintel-Höping N., Hammächer C., Kasaj A., Tamm M., Hanisch O. (2013). The gingival biotype: Measurement of soft and hard tissue dimensions radiographic morphometric study. J. Clin. Periodontol..

[10] Barriviera M., Duarte W.R., Januário A.L., Faber J., Bezerra A.C.B. (2009). A new method to assess and measure palatal masticatory mucosa by cone-beam computerized tomography. J. Clin. Periodontol..

[11] Cook D.R., Mealey B.L., Verrett R.G., Mills M.P., Noujeim M.E., Lasho D.J., Cronin R.J. (2011). Relationship between clinical periodontal biotype and labial plate thickness in vivo study. Int. J. Periodontics Restorative Dent..

[12] Lee S.-P., Kim T.-I., Kim H.-K., Shon W.-J., Park Y.-S. (2013). Discriminant Analysis for the Thin Periodontal Biotype Based on the Data Acquired From Three-Dimensional Virtual Models of Korean Young Adults. J. Periodontol..

[13] Alkan Ö., Kaya Y., Alkan E.A., Keskin S., Cochran D.L. (2018). Assessment of gingival biotype and keratinized gingival width of maxillary anterior region in individuals with different types of malocclusion. Turk. J. Orthod..

[14] Palkovics D., Gera I. (2016). The significance of biotype in the predictability of dental-periodontal treatment. Fogorv. Szle..

[15] Gonçalves Motta S., Camacho M., Quintela D., Santana R. (2017). Relationship Between Clinical and Histologic Periodontal Biotypes in Humans. Int. J. Periodontics Restorative Dent..

[16] Müller H.P. (1997). Gingival phenotypes in young male adults. J.Clin. Periodontol..

[17] Park J.H., Hong J.Y., Ahn H.W., Kim S.J. (2018). Correlation between periodontal soft tissue and hard tissue surrounding incisors in skeletal Class III patients. Angle Orthod..

[18] Rasperini G., Acunzo R., Cannalire P., Farronato G. (2017). Influence of Periodontal Biotype on Root Surface Exposure During Orthodontic Treatment: A Preliminary Study. Int. J. Periodontics Restor. Dent..

[19] Singh J., Rathod V.J., Rao P.R., Patil A.A., Langade D.G., Singh R.K. (2016). Correlation of gingival thickness with gingival width, probing depth, and papillary fill in maxillary anterior teeth in students of a dental college in Navi Mumbai. Contemp. Clin. Dent..

[20] Stellini E., Comuzzi L., Mazzocco F., Parente N., Gobbato L. (2013). Relationships between different tooth shapes and patient’s periodontal phenotype. J. Periodontal Res..

[21] Zweers J., Thomas R.Z., Slot D.E., Weisgold A.S., Van Der Weijden F.G.A. (2014). Characteristics of periodontal biotype, its dimensions, associations and prevalence: A systematic review. J. Clin. Periodontol..

[22] Cortellini P., Bissada N.F. (2018). Mucogingival conditions in the natural dentition: Narrative review, case definitions, and diagnostic considerations. J. Clin. Periodontol..

[23] Seibert J., Lindhe J. (1989). Esthetics and Periodontal Therapy. Textbook of Clinical Periodontology.

[24] Olsson M., Lindhe J., Marinello C.P. (1993). On the relationship between crown form and clinical features of the gingiva in adolescents. J. Clin. Periodontol..

[25] Aimetti M., Massei G., Morra M., Cardesi E., Romano F. (2008). Correlation between gingival phenotype and Schneiderian membrane thickness. Int. J. Oral Maxillofac. Implants.

[26] Kan J.Y.K., Morimoto T., Rungcharassaeng K., Roe P., Smith D.H. (2010). Gingival biotype assessment in the esthetic zone: Visual versus direct measurement. Int. J. Periodontics Restorative Dent..

[27] Caton J.G., Armitage G., Tonetti M.S., Papapanou P.N. (2018). A new classification scheme for periodontal and peri-implant diseases and conditions. Introduction and key changes from the 1999 classification. J. Periodontol..

[28] Olsson M., Lindhe J. (1991). Periodontal characteristics in individuals with varying form of the upper central incisors. J. Clin. Periodontol..

[29] Fu J.-H., Yeh C.Y., Chan H.L., Tatarakis N., Leong D.J., Wang H.L. (2010). Tissue Biotype and Its Relation to the Underlying Bone Morphology. J. Periodontol..

[30] Kan J.Y.K., Rungcharassaeng K., Umezu K., Kois J.C. (2003). Dimensions of peri-implant mucosa evaluation of maxillary anterior single implants in humans. J. Periodontol..

[31] Frost N.A., Mealey B.L., Jones A.A., Huynh-Ba G. (2015). Periodontal Biotype: Gingival Thickness as It Relates to Probe Visibility and Buccal Plate Thickness. J. Periodontol..

[32] Eghbali A., De Rouck T., De Bruyn H., Cosyn J. (2009). The gingival biotype assessed by experienced and inexperienced clinicians. J. Clin. Periodontol..

[33] Claffey N., Shanley D. (1986). Relationship of gingival thickness and bleeding to loss of probing attachment in shallow sites following nonsurgical periodontal therapy. J. Clin. Periodontol..

[34] Pontoriero R., Carnevale G. (2001). Surgical crown lengthening 12-month clinical wound healing study. J. Periodontol..

[35] Hwang D., Wang H.-L. (2006). Flap Thickness as a Predictor of Root Coverage: A Systematic Review. J. Periodontol..

[36] Wennström J.L., Lindhe J., Sinclair F., Thilander B. (1987). Some periodontal tissue reactions to orthodontic tooth movement in monkeys. J. Clin. Periodontol..

[37] Kao R.T., Fagan M.C., Conte G.J. (2008). Thick vs. thin gingival biotypes key determinant in treatment planning for dental implants. J. Calif. Dent. Assoc..

[38] Evans C.D.J., Chen S.T. (2008). Esthetic outcomes of immediate implant placements. Clin. Oral Implants Res..

[39] Cosyn J., Eghbali A., De Bruyn H., Collys K., Cleymaet R., De Rouck T. (2011). Immediate single-tooth implants in the anterior maxilla: 3-year results of a case series on hard and soft tissue response and aesthetics. J. Clin. Periodontol..

